# Incidental detection of operational tolerance in a deceased donor kidney transplant recipient lost to follow-up for more than 10 years: A case report and literature review

**DOI:** 10.5414/CNCS111030

**Published:** 2023-02-16

**Authors:** Sasmit Roy, Monzurul Hasan Chowdhury, Debargha Basuli, Sreedhar Adapa, Kenneth Bodziak

**Affiliations:** 1 University of Virginia, Lynchburg Dialysis Unit, Lynchburg, VA,; 2 Frank H. Netter M.D. School of Medicine, Department of Internal Medicine, North Haven, CT,; 3 Department of Internal Medicine, East Carolina University, Greenville, NC,; 4 Kaweah Delta Medical Center, Visalia, CA, and; 5 Department of Nephrology, Banner University Medical Center, Phoenix, AZ, USA

**Keywords:** operational tolerance, graft survival, kidney transplantation, immunosuppression medications

## Abstract

Graft tolerance is a clinical state of absence of an immune response in the recipient toward a donor allograft without any exogenous immunosuppression. Although more prevalent in liver transplantation recipients, it has rarely been reported in renal transplant recipients. We present a 62-year-old deceased donor kidney transplant recipient who exhibited operational tolerance as they stopped immunosuppressant medications for more than 10 years and yet demonstrated stable graft function. Although various hypotheses, such as deletion, anergy, immunoregulation, and clonal exhaustion, have been experimentally validated, clinical “operational tolerance” of a renal allograft on a prolonged basis has been infrequently reported in the medical literature. This review intends to highlight possible etiologies and make clinicians aware of this possible rare condition to which more research is needed.

## Introduction

Graft tolerance is a clinical entity perceived as stable allograft function in the absence of any immunosuppressive drugs, while without any clinical characteristics of chronic rejection and is observed minimally for 1 year [1]. Though observed more frequently in liver transplant patients than in other solid-organ recipients [2], report of spontaneous graft tolerance in kidney allograft recipients has rarely been noticed [1].

Physicians often detect these instances serendipitously while treating patients for other non-transplant-related ailments. Further inquiry into these patients reveals that not taking immunosuppressant medications still maintains a steady graft function. Although various hypotheses, such as deletion, anergy, immunoregulation, and clonal exhaustion, have been experimentally validated, clinical “operational tolerance” of a renal allograft on a prolonged basis has been infrequently reported in the medical literature. We report a similar case of immune tolerance following a deceased donor kidney transplant in a patient with normal allograft function who remained disease-free for over 10 years without being on immunosuppression medications.

## Case report

**Figure 1.**
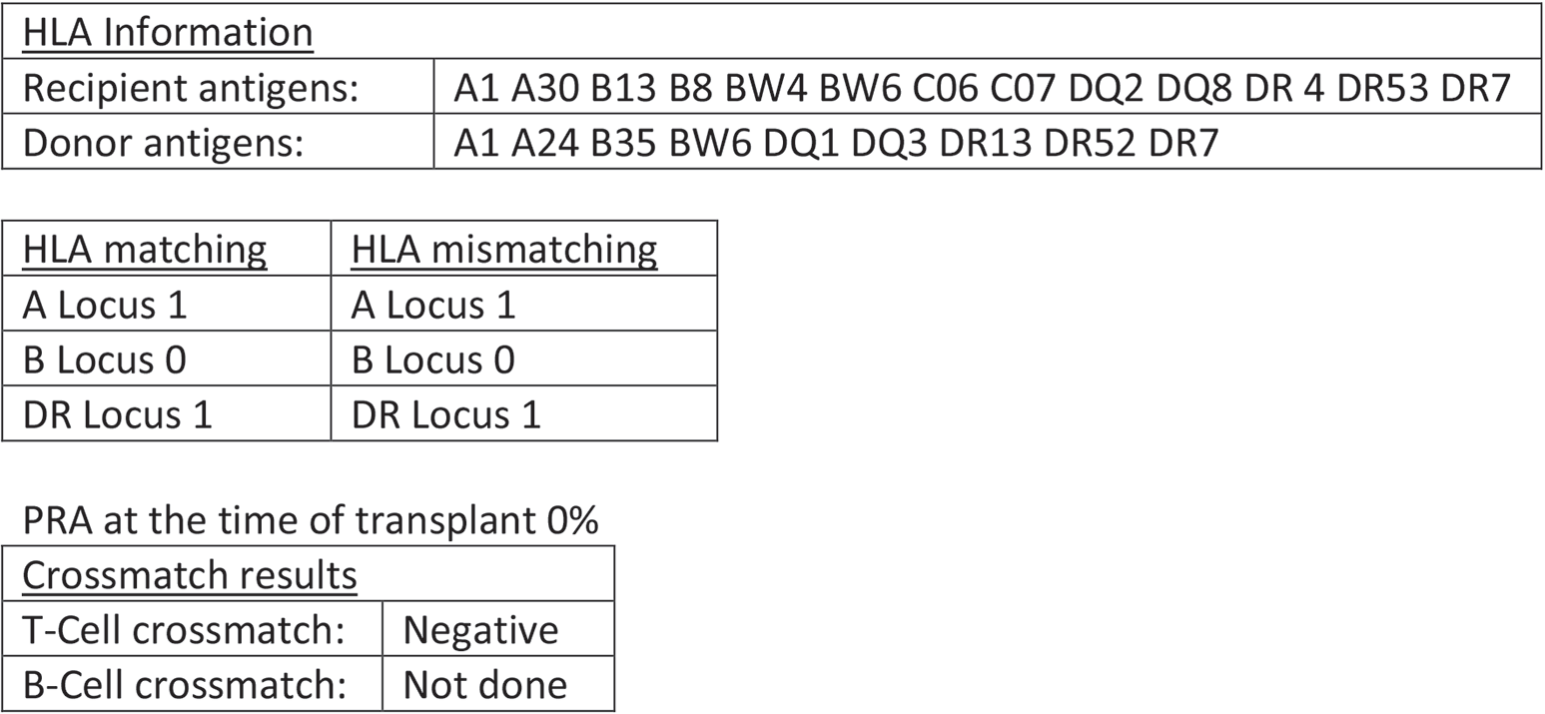
Transplant laboratory data.

The patient was a 62-year-old White male with a history of a deceased donor kidney transplant 16 years ago. He presented to the hospital with complaints of diarrhea, fever, and abdominal pain for 7 days. His past medical history included end-stage renal disease (ESRD) attributed to diabetes mellitus type II, hypertension, recurrent nephrolithiasis with left nephrectomy after a staghorn calculus, recurrent urinary tract infections, and alcohol dependence. He initially started continuous ambulatory peritoneal dialysis (PD). After 4 years of PD initiation, he was converted to hemodialysis (HD) because of PD failure. After 1.5 years on HD, he received an ABO-compatible deceased donor kidney transplant. His panel-reactive antibodies (PRA) pre-transplant were 0%. Human leukocytic antigen (HLA) matching was 2/6, with one match each at A**-** and DR– locus (Figure 1). Flow cytometry showed negative T-cell and B-cell crossmatch.

The donor was CMV-positive, and the recipient was CMV-negative. Initial immunosuppression induction agents were intravenous (IV) rabbit anti-thymocyte thymoglobulin at 2 mg/kg and IV methylprednisolone at 7 mg/kg intraoperatively. The induction regimen was followed by 2 mg/kg of IV thymoglobulin for the following 2 days. His postoperative course was uncomplicated without any delayed graft function. His serum creatinine improved from 5 to 2.0 mg/dL at discharge time on postoperative day 5. Maintenance immunosuppression consisted of tacrolimus, mycophenolate mofetil, and prednisone. Prednisone was started at 1 mg/kg daily for the first week and tapered down to 10 mg daily by 4 weeks. Tacrolimus was started at 0.08 mg/kg with a target 12-hour trough of 7 – 10 ng/mL in the first month and a subsequent 4 – 6 ng/mL target level. Mycophenolate mofetil was started at 500 mg twice daily. Within the first year of the transplant, he moved to a different state for job-related issues, gradually forgot to keep up with his follow-up appointments, and eventually, after a year, stopped taking all his medications, including the immunosuppressants. His last creatinine before being lost to follow-up was 1.5 mg/dL.

The patient presented 15 years post-transplant to our hospital for an unrelated acute abdominal pain medical event. During this admission, initial physical examination vitals were blood pressure 101/65 mm Hg, heart rate 77/min, temperature 99.3 F (37.4 °C), oxygen saturation 99% room air. Physical examination was positive for mild tenderness on palpation in the right lower quadrant, but without any guarding or distension, and with affirmative bowel sounds. The rest of the physical examinations were unremarkable, including cardiovascular, respiratory, and neurological systems. His serum creatinine on presentation was 1.19 mg/dL, and his urine was negative for proteinuria. Other laboratory findings were as shown in Table 1. Computed tomography (CT) of the abdomen and pelvis showed a perforated appendix with the surrounding fluid. Ultrasound of the transplanted kidney did not reveal any evidence of decreased flow, stenosis, or localized fluid collection in the allograft. The patient was empirically started on IV broad-spectrum antibiotics – vancomycin and piperacillin-tazobactam – and then taken to the operating room for drainage of the abscess. He recovered from their current illness, and on discharge his serum creatinine was 1.07 mg/dL with eGFR 75 mL/min.

It was appreciated that he had preserved his renal function for all these years without immunosuppressive medications. He had not received any blood transfusions or major surgeries in the prior 15 years. A transplant biopsy was deferred in the setting of normal graft function. Donor-specific antibody (DSA) test was positive for DR13 only with a mean fluorescent intensity (MFI) of 14000, with no other reported presence of preformed or de novo antibodies in the DSA test. Donor-derived cell-free DNA testing was unavailable in our facility at the time of presentation. He was subsequently discharged without any immunosuppressants. Two years post-hospitalization, he still demonstrated stable graft function, with the most recent creatinine level being 1.25 mg/dL in the absence of all immunosuppressive medications. His urine protein creatine ratio remained unremarkable, and urine analysis stayed bland.

## Discussion

Initially described by Billingham et al. [3] in 1953 in a mouse, tolerance to transplantation is an acquired condition of recipient unresponsiveness towards donor-specific antigens. The widespread advantage of preventing acute graft rejection by current immunosuppressant medications is acknowledged worldwide. However, the cumulative toxicity and pill burden deter graft survival and patient compliance. Chronic allograft nephropathy can be heralded by immune-related (actual rejection) or non-immune-related reasons (immunosuppressive drug toxicity and adverse effects like hyperlipidemia, diabetes, primary disease recurrence, and ischemia/reperfusion injury) [4, 5].

“Operational tolerance” is a clinical entity with stable allograft function in patients taking no immunosuppressive drugs, without any clinical characteristics of chronic rejection, and persisting for at least more than 1 year [6]. In an extensive review article of different collaborative studies, Massart et al. [6] described that at least 1 year of stable kidney function is required to define this term of operational tolerance. The acceptable threshold for stable graft function has been concluded as serum creatinine level < 1.5 mg/dL and proteinuria < 1 gm/24 hours [1]. Evolutionary tolerance to donor alloantigen in vivo involves many mechanisms attributed at different stages [7]. The persistence of alloantigen is believed to be fundamental for most of the mechanisms reported to be involved in experimental rodent studies, namely deletion, clonal exhaustion, ignorance, anergy, and immunoregulation. Without the alloantigen, tolerance is nullified either gradually or immediately [8, 9]. Deletion works by obliterating donor-alloantigen-reactive T cells either centrally in the thymus [10], maintained only transiently, or peripherally in the blood, where alloantigen recognition is done under suboptimal conditions [11]. On the other hand, anergy works by functionally inactivating the T-cell response to alloantigen restimulation. Clonal exhaustion occurs due to chronic alloantigen stimulation or recognition under suboptimal conditions. Repeated exposure leads to gradual cell exhaustion. This method is widespread in liver transplantation [12]. Immunoregulation is an ongoing process where one population of cells regulates the activity of the other population. A population of T cells referred to as regulatory T cells play a pivotal role in inhibiting an immune reaction against the body’s self-antigens. The secretion of immunosuppressive cytokines, such as TGF-β and IL-10, which inhibit lymphocyte activation and effector functions, may mediate these inhibitory effects of the regulatory T cells [7].

Due to its rare occurrence, data on patients’ clinical characteristics in operational tolerance is limited. In one study by Roussey-Kesler et al. [1], it was demonstrated that out of 10 patients studied for operational tolerance, the mean age was 9.4 years, and the median donor age was 25 years, suggesting good graft quality. Although transiently, mixed chimerism has been achieved through various protocols by induction of tolerance in HLA‐mismatched human kidney recipients [13, 14]. Mixed chimerism refers to a “hybrid immune system” where hematopoietic lineage in the recipient is achieved when donor hematopoietic pluripotent stem cells coexist and engraft with the recipient’s stem cells [15]. Upon successful engraftment, recipient immune cells establish a long sustainable acceptance of the same donor’s tissue and organ transplants [16]. In our patient, a chimerism study was sent from peripheral blood and returned negative. However, we could not exclude microchimerism based on this test alone (Table 2).

Combining bone marrow infusion from the kidney donor and various immunomodulating or lymphodepleting chimerism in the recipient can be achieved through different regimens. An increasing number of research groups have embarked on an effort to develop cellular therapies to achieve more durable and lasting allograft tolerance [17]. In > 40 years of case reports, only 247 cases of clinical tolerance have been reported. Of them, hardly 15 – 20 patients have sustained clinical tolerance for > 10 years [6]. The largest of the trials done so far, the DESCARTES-Nantes wide-scale European survey, where 40 patients were studied, suggested that the donors were relatively young, and that there was no difference in baseline characteristics of operationally tolerant kidneys. Out of 35 newly operational tolerant patients finally identified, the mean age of recipients was young at 29 years, the mean age of donors was young at 33.5 years, and the mean period of optimal graft function without immunosupressants was 70 months [18]. There was also the occasional presence of anti-donor HLA antibodies [18]. Most successful cases of clinical tolerance have been reported in living donor recipients [19].

Thus, although successfully demonstrated in experimental studies, “Operational tolerance” remains an infrequent event in clinical transplantation, primarily attributed to luck or mere coincidence. Conditions limiting the extensive search for clinical transplantation tolerance might be the absence of validated assays or biomarkers conducive to tolerance, the dearth of therapeutic protocols favored for tolerance induction in humans, and genuine worry about the ethics and safety of total withdrawal of immunosuppression in this age of widely successful immunosuppressive medications. Although still in the nascent stage of clinical validity, B cells isolated from tolerant patients seem to play an essential role in tolerance induction through inhibiting CD4 1 CD25-effector T cells [20]. In contrast to rejecting and immunosuppressed patients, rejection-free stable kidney recipients and operationally tolerant patients have demonstrated an increase of transitional and naïve B-cell clones with their associated transcripts [21].

Our patient gradually discontinued his immunosuppressive medications alone without any documented side effects or worsening of his graft function. Although the exact mechanism remains unknown, possible mechanisms for his immune tolerance could be central (intrathymic) deletion, peripheral ignorance, anergy, and microchimerism, either alone or in combination. As of this day, he still maintains stable graft function without requiring the support of any immunosuppressant medications.

## Conclusion

Organ transplantation typically requires prolonged immunosuppressant use to ensure viable allograft survival. Often, this comes with issues of serious adverse effects and, thereby, patient compliance. Presently, the exact mechanism for developing operational tolerance remains to be elucidated. There is a dire need to increase awareness of this condition and research the tolerance factors in these patients. Hopefully, continuous monitoring of patients who have achieved spontaneous tolerance might improve our knowledge of the underlying tolerance mechanism.

## Funding

There was no funding for this report.

## Conflict of interest

The authors have no conflict of interest to report.

**Table 1.** Laboratory findings on admission day.

Laboratory parameters	Value	Reference range
Sodium	129 mmol/L	135 – 145 mmol/L
Potassium	4.2 mmol/L	3.5 – 5.1 mmol/L
Chloride	96 mmol/L	95 – 105 mmol/L
CO_2_	20 mmol/L	22 – 33 mmol/L
Blood urea nitrogen (BUN)	17 mg/dL	8 – 20 mg/dL
Creatinine	1.19 mg/dL	0.6 – 1.3 mg/dL
eGFR (glomerular filtration rate)	65 mL/min/m^2^	> 60 mL/min/m^2^
Calcium	8.5 mg/dL	8.5 – 10.5 mg/dL
Albumin	2.6 gm/dL	3.5 – 5 gm/dL
Glucose	143 mg/dL	70 – 105 mg/dL
Hemoglobin	13.7 gm/dL	13.2 – 17.1 gm/dL
Hematocrit (HCT)	38.4%	15 – 45%
White blood count	12.5 × 10^9^/L	4 – 11 × 10^9^/L
Urine protein	Absent	–
Urine blood	Absent	–

**Table 2.** Chimerism study by STR-PCR method.

Sample Local Id	Sample identifier	Sample type	Collected	Testing date	Donor %	Comment
611183-2		Buccal swab	March 8, 2019	March 15, 2019	< 3%	No donor DNA detected
611183-1		Post-transplant blood	March 2, 2019	March 7, 2019	< 3%	No donor DNA detected

Threshold for positivity > 3%.
